# A77 THE ASSOCIATIONS OF OBJECTIVELY ASSESSED SEDENTARY TIME AND STEP COUNT ON ULCERATIVE COLITIS OUTCOMES

**DOI:** 10.1093/jcag/gwac036.077

**Published:** 2023-03-07

**Authors:** B A Chiew, K A Lyden, A Schick, C Ohland, K McCoy, S Kaur, M Yousuf, L Taylor, M Raman, J K Vallance

**Affiliations:** 1 Cumming School of Medicine, University of Calgary, Calgary, Canada; 2 KAL Research & Consulting, LLC, Denver, United States; 3 University of Calgary, Calgary, Canada; 4 Department of Physiology and Pharmacology, Snyder Institute of Chronic Disease, Cumming School of Medicine; 5 International Microbiome Centre, Cumming School of Medicine, University of Calgary; 6 Faculty of Health Disciplines, Athabasca University, Calgary, Canada

## Abstract

**Background:**

Physical activity has been associated with positive health outcomes in those with Ulcerative Colitis (UC). The extent to which other more prominent behaviours occurring throughout the 24-hour day (i.e., sitting, standing, lying down, and stepping) are associated with UC outcomes is unknown.

**Purpose:**

The purpose of this study was to explore whether objectively measured time spent sitting, lying down, standing, and stepping were associated with Total Mayo score (TMS), fecal calprotectin (FCP), and C-reactive protein (CRP) in patients with UC.

**Method:**

Patients were recruited from the Foothills Medical Center in Calgary, Alberta and were given activPAL^TM^ accelerometers (PAL Technologies Limited, Glasgow, UK) to wear on their thigh for 7 days. Step count, sitting time, standing time, and time lying down (excluding sleep) were recorded for a minimum of 4 days, including at least one day on the weekend. TMS was used to determine disease activity and patients were categorized into normal/mild (TMS score <6) or moderate/severe (TMS score 6). FCP, a marker of gut inflammation, was measured using stool samples. Blood samples were collected to measure serum CRP, a marker of systemic inflammation. Univariate analysis of covariance (ANCOVA) was used to evaluate associations between the activPAL^TM^ daily activity variables, TMS, FCP (< or >250ug/g) and CRP (< or > 5 mg/L). Analyses were controlled for age, sex, body mass index (BMI), and antibiotic use.

**Result(s):**

Patients (N=29; 15 male, 14 female) were on average 38 years of age (SD=12.1). The average BMI was 26.2 kg/m^2^ (SD=3.2). Based on TMS, 14 had moderate/severe disease activity and 16 had normal/mild disease activity. Average CRP was 2.16 mg/L (SD=2.49) while the mean FCP was 954.5 ug/g (SD=1427.7). Patients recorded an average of 8,137 steps (SD=3,051) per day. Average standing time was 240 minutes (SD=84) per day, sitting time was 503 minutes (SD=131) per day, and time spent lying down was 527 minutes (SD=111) per day. FCP was negatively associated with step count (D=-2,134 steps, 95% CI: -4,360 to 93, p=0.06). Patients with lower FCP values (<250mg/g) spent 60 fewer minutes sitting (p=.25), and 52 more minutes standing (p=.12) during the day compared to patients with higher FCP values (>250mg/g). Patients with normal/mild disease severity (TMS <6) spent 83 fewer minutes per day sitting compared to those with moderate/severe disease severity (TMS >6, p=.12). CRP was not associated with any behavioural outcomes.

**Conclusion(s):**

In our study, daily steps appeared to be most strongly associated with FCP. While not statistically significant, patients with lower FCP reported less sitting and more standing compared to those with higher FCP. Future studies with larger sample sizes should continue to explore these activity behaviours and their potential associations with UC disease outcomes.

**Please acknowledge all funding agencies by checking the applicable boxes below:**

Other

**Please indicate your source of funding;:**

Alberta's Collaboration of Excellence for Nutrition in Digestive Diseases (ASCEND)

**Disclosure of Interest:**

B. Chiew: None Declared, K. Lyden Consultant of: PAL Technologies, the company that manufactures the activPAL device., A. Schick: None Declared, C. Ohland: None Declared, K. McCoy: None Declared, S. Kaur: None Declared, M. Yousuf: None Declared, L. Taylor: None Declared, M. Raman Shareholder of: LyfeMD – Director, Shareholder, Grant / Research support from: Pfizer, Takeda, Speakers bureau of: Fresenius Kabi, Pfizer, Mckesson, Takeda, Lupin, J. Vallance Grant / Research support from: Canada Research Chairs Program

